# Everybody's Page

**Published:** 1889-08-24

**Authors:** 


### Everybody's Page.

A druggist recently received a note asking him to send "sixpennyworth of Senia and Mania, with a little Aniseed in it."

---

The demand for low-priced wine has tended largely to make Spanish wine-growers use industrial alcohol. Those who continue to make wine purely from the grape cannot compete with those who adulterate it with potato-spirit, and make the difference less apparent by colouring matter of various kinds.

---

In Egypt the temples of Saturn, and in Greece the hospitals erected in honour of Æsculapius were used as asylums for the insane, and the treatment there followed was the same in principle as that of the present day. The advice given by Hippocrates and Galen for the treatment of lunacy is such as to satisfy the *aliénistes* of our own time.

---

A chutnee, which will bear comparison with that imported, may be made from gooseberries. The recipe is as follows: Ingredients: 2 quarts of gooseberries, 2 quarts of vinegar, 1lb. salt, 1lb. mustard seed, 1lb. stoned raisins, 1lb. brown sugar, 12oz. garlic, 6oz. cayenne. Make a syrup of the sugar with a pint of vinegar; boil the gooseberries in the remainder of it; bruise the mustard seed and garlic, and mix all the ingredients thoroughly in a mortar.

---

It is quite a mistake to suppose, as many people do, that a "Government stamp" is any guarantee of the merit of a patent medicine. It simply means that the State demands a certain percentage on the sale of the medicine. A stamp duty of this sort is a very dubious gain to the nation, for, though it puts a certain amount of money into the Treasury, it gives a dangerous *prestige* to compounds that are not always innocuous, and tempts people to buy drugs that may be, for them, and taken without medical supervision, nothing less than poisonous.

---

Sponge fishing is carried on along the coast of Tripoli, Latakia, Batroon, and Ruad, a small island to the north of Tripoli. The fishers are chiefly Greeks or Syrians, and their stay under water is usually limited to sixty or eighty seconds. They cannot be persuaded to adopt the diving dress, which would enable them to stay under water for hours. The average depth to which they descend is 25 to 175 feet, below which no good sponges are to be found. The annual value of the catch is about £30,000, from which the authorities deduct a tax of 10 per cent.

---

Dr. Lewis H. Adler, of the Pennsylvania University Hospital, has come to the conclusion, from the study of the amputations that have taken place there during the last fourteen years, that amputations of the lower extremity are more liable to prove fatal than those of the upper extremity; that the rate of mortality is greater in proportion to the proximity of the part amputated to the trunk; and that, contrary to the general belief, the mortality of amputations for chronic disease is about as large as for recent injuries, in both being about 24 per cent.

---

Olive-seed is much used in France as an adulterant for pepper. It is a regular article of trade, and is exported from Marseilles. It is supplied, like the pepper, in black and white, the white being produced by peeling the seed before grinding it. This adulteration is not easily detected by the microscope. Another popular addition is roasted breadcrumbs. It is very difficult for detectives to get ahead of the adulterators on any point. They have chemists in their employ, who not only concoct mixtures for them, but are on the alert to study any new books on adulterations that are published.

---

Badly made coal gas contains 20 or 30 per cent. of carbonic oxide, which acts as a poison by taking the place of oxygen in the red blood corpuscles.

---

The expressive word "booze" is derived from "bouza," an intoxicant made from millet seeds with some powerful astringents added, which is the favourite drink of the nomadic tribes of Tartary.

---

One of the worst results of the use of alcohol as a beverage is that it makes it useless as a drug. In some diseases alcohol is a valuable remedy, but in persons who are habituated to its use the system fails to respond to the stimulating action of the drug, a failure which may make all the difference between death or recovery.

---

Carbolate of camphor is recommended as a dressing for wounds. It is made by dissolving camphor in a 95 per cent. solution of carbolic acid to the point of saturation. The product tastes and smells more of camphor than carbolic, and when mixed with an equal quantity of cotton-seed oil and applied to a wound on gauze and cotton it prevents suppuration.

---

Personal health and national health are not quite the same thing in different sizes. A man may be called healthy who lives in comfortable idleness, is able to gratify his wishes without suffering afterwards, and dies painlessly in old age. But a nation whose members attained only such comfortable animalism as this, would not be in a healthy condition. In view of national health, every man must produce good work besides looking after his own comfort, and even, perhaps, in preference to that.

---

One firm alone in Newark, New Jersey, makes a hundred and fifty millions of corkscrews every year. Now a corkscrew is not a thing that wears out rapidly, and a recent writer, who has collected the statistics of the manufacture, is at a loss to understand how the demand year by year equals the supply. In Newark alone enough corkscrews are manufactured to supply every man, woman, and child in Europe and America with one corkscrew a year. "What becomes of the old ones?" he asks. "Is there a demand for second-hand corkscrews in Central Africa?"

---

One can't help wondering sometimes how all the girls who would make such admirable wives remain unmarried, and have to sigh in loneliness over the incompetence of their matron friends. One of these unappropriated blessings, an American by nationality, has been sighing in print over the extravagance of the American housewife. That degraded being throws away 5,000,000 dols. annually on "bluing." (Of course we are taking the housewife as a national entity, and don't condescend to enquire how many pence per household this amounts to. You can't get imposing effects that way.) "Bluing," we are told, is useless. Well, yes, but one doesn't like to have all one's linen go yellow, as it will after repeated washings, unless this same unnecessary "bluing" is allowed to have its counteracting effect. Why doesn't the complainant find fault with the use of starch. It costs a great deal more per annum than blue does, and, moreover, it is a waste of potential food. We ourselves feel quite equal to the task of getting up a crusade against starch, and making the most touching appeals to the public to refrain from using it. But we know, and perhaps the fair American economist knows too, that while paterfamilias doesn't, as a rule, mind his shirts and collars being "a bad colour," as his wife calls it, he will be very disagreeable if they are not stiff enough. It is much safer to quarrel with the "bluing."

